# Compounded Effects of Multiple Global Crises on Mental Health: A Longitudinal Study of East German Adults

**DOI:** 10.3390/jcm13164754

**Published:** 2024-08-13

**Authors:** Ernst Peter Richter, Elmar Brähler, Markus Zenger, Yve Stöbel-Richter, Franziska Emmerich, Julia Junghans, Juliana Krause, Lisa Irmscher, Hendrik Berth

**Affiliations:** 1 Research Group for Applied Medical Psychology and Medical Sociology, Division of Psychological and Social Medicine and Developmental Neurosciences, Faculty of Medicine, TU Dresden, 01307 Dresden, Germanyfranziska.emmerich@mailbox.tu-dresden.de (F.E.); julia.junghans@mailbox.tu-dresden.de (J.J.); lisa.irmscher@ukdd.de (L.I.); 2 Department of Psychosomatic Medicine and Psychotherapy, University Medical Center of the Johannes Gutenberg University, 55131 Mainz, Germany; elmar.braehler@medizin.uni-leipzig.de; 3 Department of Psychiatry and Psychotherapy, University Medical Center of the University, 04103 Leipzig, Germany; 4 Faculty of Applied Human Studies, University of Applied Sciences Magdeburg and Stendal, 39576 Stendal, Germany; markus.zenger@h2.de; 5 Integrated Research and Treatment Center Adiposity Diseases—Behavioral Medicine, Psychosomatic Medicine and Psychotherapy, University Medical Center of the University, 04103 Leipzig, Germany; 6 Faculty of Managerial and Cultural Studies, University of Zittau/Goerlitz, 02826 Goerlitz, Germany; yve.stoebel-richter@hszg.de

**Keywords:** mental distress, life satisfaction, sleep problems, COVID-19, Russia–Ukraine-War, climate change, longitudinal study, public health, hierarchical partitioning

## Abstract

The early 2020s witnessed an unprecedented overlap of multiple global crises. This longitudinal study examined the compounded effects of multiple intersecting global crises on mental health outcomes in a representative cohort of East German adults. We investigated how perceived threats (PT) from climate change (PT-CLC), COVID-19 (PT-COV), the Russia–Ukraine War (PT-RUW), and rising costs of living (PT-RCL) will impact various aspects of mental health from 2021 to 2022. This research question addresses whether these crises exacerbate mental health issues and how their effects differ across various mental health outcomes. **Methods:** We conducted a longitudinal study with 319 participants (mean age 49.9 years, 54.5% female) from the Saxony Longitudinal Study. Data were collected in two waves: March–July 2021 and September–December 2022. We used linear mixed-effects models to analyze both unadjusted group trends and adjusted individual-level effects on physical complaints, mental distress, sleep problems, life satisfaction, and self-rated health. **Results:** Unadjusted analyses revealed significant increases in mental distress and sleep problems over time, whereas physical complaints, life satisfaction, and self-rated health remained stable at the group level. Adjusted analyses showed that higher PT-RCL and PT-COV were significantly associated with increased physical complaints, mental distress, sleep problems, and decreased life satisfaction, even when group-level changes were not significant. **Conclusions:** This study highlights the complex impact of intersecting global crises on mental health, emphasizing the importance of considering both population-level trends and individual perceptions. The findings suggest that economic and pandemic-related stressors have more immediate effects on mental health outcomes compared to more distant threats, such as climate change or geopolitical conflicts.

## 1. Introduction

During the early 2020s, multiple global crises overlapped, profoundly affecting daily life. Global crises are large-scale complex challenges that can transcend national borders, disrupt social and economic systems, and pose severe threats to human well-being [1]. Research has shown that even a single global crisis, such as a natural disaster [2] or terrorism [3], can adversely affect mental health. Extensive media coverage of these crises, featuring vivid images and reports of trauma, can evoke anxiety, sadness, and stress, leading to persistent distress [4,5]. Furthermore, global crises create ambiguity and anxiety about the future, causing individuals to worry about the stability of their lives and communities [6,7].

To gain insight into the concerns of the German population, the Security Report, an annual nationwide survey by the Allensbach Institute, assessed the perceptions of various threats and security concerns among German citizens aged 16 years and above. In January 2021, 45% of East German respondents expressed significant concerns about Germany’s potential involvement in military conflicts, 53% were concerned about managing the COVID-19 crisis, 44% were apprehensive about climate change, and 41% were worried about inflation [8]. By January 2023, concerns about inflation had escalated dramatically, with 88% of respondents worried about rising costs of living. In addition, 87% expressed deep concerns regarding the ongoing conflict in Ukraine. In contrast, concerns about climate change decreased to 39% and apprehensions regarding COVID-19 sharply declined to 13% [9].

The importance of capturing these concerns lies in understanding the psychosocial dynamics that underlie a population’s response to crises. The perception of multiple overlapping global threats can significantly exacerbate mental health issues, particularly in a nation like Germany, where historical experiences and current socioeconomic factors intersect. By analyzing how different segments of the population, such as East Germans, perceive and react to these crises, researchers can identify key stressors that influence mental health outcomes. Such insights are critical for informing public health policies and interventions aimed at mitigating the psychological impacts of ongoing and future crises. The following sections provide an overview of the mental health consequences associated with each crisis.

### 1.1. Climate Change

Climate change can have a significant impact on mental health, leading to distress, anxiety, depression, and increased levels of posttraumatic stress disorder [10,11]. Research has focused primarily on the mental health consequences of exposure to extreme weather events such as high temperatures, tornadoes, and wildfires [12]. However, climate change exposure in Germany is often indirect and occurs primarily through media portrayals of environmental challenges [13]. Media reports can generate feelings of uncertainty and stress, contributing to depression and helplessness. A study across 25 countries provided conclusive evidence of a relationship between negative emotions related to climate change and poor mental health, including insomnia symptoms and a lower self-rated mental state [14].

### 1.2. The COVID-19 Pandemic

SARS-CoV-2 spread rapidly in early 2020, causing a significant number of deaths. Beyond its immediate health consequences, the pandemic has inflicted considerable psychological stress. Individuals face challenges such as adapting to remote work and online learning, managing economic instability, and fear of contracting the virus [15]. During the pandemic, the prevalence of stress, anxiety, and depression in the general population increased by 29.6%, 31.9%, and 33.7%, respectively [16]. Emotional reactions vary within a population and are influenced by individual coping strategies, pre-existing psychological conditions, and contextual factors [17]. Regional differences in emotional distress levels suggest disparities in healthcare infrastructure and public health policies [18].

**Figure 1:**
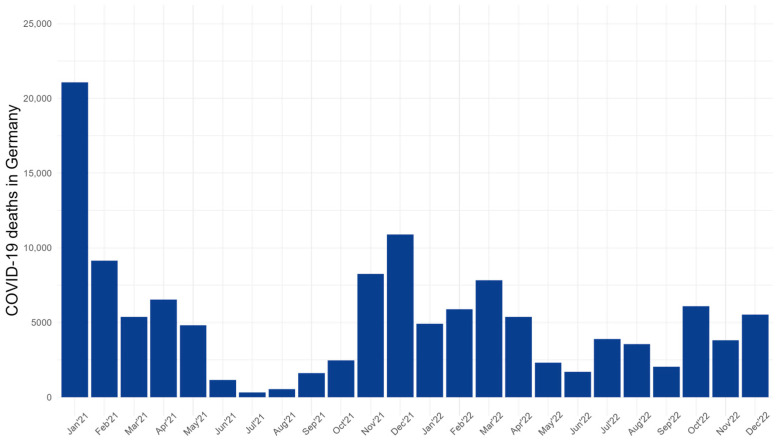
COVID-19 deaths in Germany from January 2021 to December 2022.

As illustrated in Figure 1, the trend of COVID-19 mortality rates in Germany initially showed high trends from spring 2021 to autumn 2022 and these rates increased again during the subsequent waves of the pandemic despite increasing vaccination rates. By the end of 2022, fatalities had significantly decreased, attributable to high vaccination coverage and reduced severity of the prevailing viral strains [19].

### 1.3. Russia–Ukraine War (RUW)

Concurrent with ongoing global efforts to address climate change and mitigate the repercussions of the COVID-19 pandemic, an armed conflict commenced in Ukraine on 24 February 2022. Within four months, more than 0.9 million Ukrainian refugees entered Germany. The war and refugee influx led to significant societal changes in Germany, affecting economic and political dynamics and causing profound psychosocial consequences [20].

The DigiHero study, involving over 19,400 participants from Saxony-Anhalt, Saxony, and Bavaria, revealed elevated levels of anxiety and depression in the weeks following the war’s onset and six months later. Although anxiety and distress decreased over time, symptoms remained clinically significant six months later [21,22].

A study involving 1300 individuals across 17 European countries revealed an acute decline in well-being on the day of invasion, with recovery to previous levels in subsequent weeks. Social media intensified daily distress by frequently highlighting war in the news, highlighting the substantial impact of geopolitical events on psychological well-being. Personality traits also influence recovery rates, suggesting that individual differences play a role in coping with such crises [23].

### 1.4. Rising Costs of Living Crisis

The Russia–Ukraine War triggered significant global financial disruptions, impacting supply chains and causing fluctuations in energy and commodity prices [24]. This has led to a substantial increase in living costs, particularly for German citizens.

**Figure 2:**
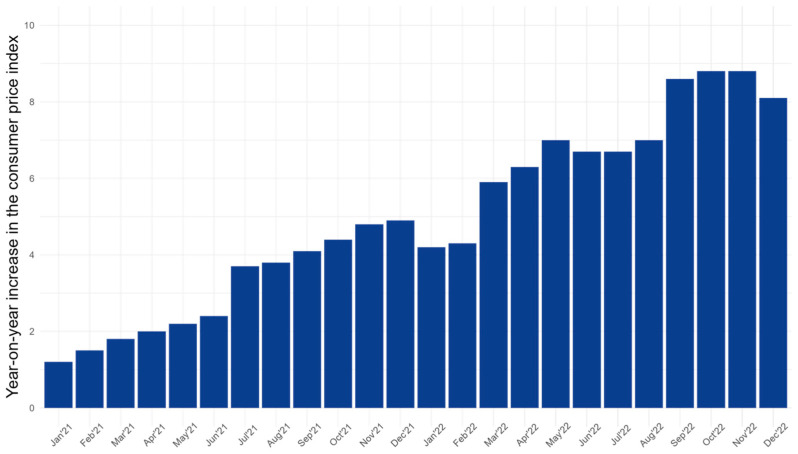
Year-on-year increase in the consumer price index in %.

Figure 2 illustrates the monthly variations in the consumer price index compared to the same month of the previous year. The inflation rate quadrupled from approximately 2% to 8% between spring 2021 and autumn 2022 [25].

Previous research has highlighted the detrimental effects of economic stressors on mental health, noting their association with heightened levels of depression, anxiety, and anger. Inflation-induced financial strain can also disrupt daily life and long-term plans, exacerbate stress, and reduce overall well-being [26,27].

A recent study demonstrated that older adults who perceive moderate to large increases in the cost of living experience greater levels of psychological distress, including symptoms of depression, anxiety, and anger. The study also revealed that a sense of mastery or control over one’s life can buffer these adverse effects, reducing the psychological impact of financial stressors [28].

A vital feature of the four global crises is their simultaneous occurrence. Existing research has primarily focused on the isolated effects of individual crises without exploring their cumulative impact.

This study addressed this limitation by evaluating and comparing perceived concerns regarding four global stressors and by examining their collective and individual impacts on mental health in a representative age-homogeneous East German cohort sample. Specifically, we sought to quantify and compare perceived levels of concern regarding each crisis and elucidate their effects on critical mental health indicators, including physical complaints, mental distress, sleep problems, life satisfaction, and self-rated health.

## 2. Materials and Methods

### 2.1. Participants

The Saxony Longitudinal Study (SLS) began in 1987 in the former German Democratic Republic (GDR). Initially, it included a cohort of 1281 14-year-old students representing the East German Republic population born in 1973, all of whom were eighth graders across 41 schools. Following the third wave in the spring of 1989, 587 individuals agreed to continue participating. The study persisted after German reunification and data were collected almost annually to the present day.

The SLS has primarily addressed political and social questions, focusing on the long-term adaptation of GDR citizens, their reactions to reunification, and subsequent changes in their living circumstances. Since 2002, the scope of this study has expanded to include physical and mental health metrics [29,30]. New focal areas and survey instruments were introduced in each wave. The study protocol (No. EK8012011) was approved by the Ethics Committee of TU Dresden and adhered to national regulations and the Declaration of Helsinki.

In the 32nd wave (W32) (March to July 2021, pre-RUW, *N* = 320 participants) and the 33rd wave (W33) (September to December 2022, post-RUW onset, *N* = 319 participants) of the study, the same instruments were used, with 289 individuals participating in both waves. The average age of the participants in the 33rd wave was 49.9 years (*SD* = 0.6), with 54.5% being female (*N* = 145) and 80.4% (*N* = 254) reporting having children. Detailed sociodemographic characteristics are shown in Table 1.

### 2.2. Measures

#### 2.2.1. Outcome Measures

Physical complaints were assessed using the G-Score questionnaire, which was developed to capture self-reported physical health complaints over a 12-month period. The G-Score comprises four items, each focusing on a common physical health issue: nervousness, stomach issues, insomnia, and heart-related concerns. Participants indicated how frequently they experienced each of these issues within the past year, with responses ranging from “frequently” to “never”. Higher scores indicate a greater burden of physical complaints. Previous studies have reported that the G-Score has satisfactory reliability [31], with a McDonald’s Omega of 0.750 in wave 33.

Psychological distress was assessed using the D-Score questionnaire, which was developed to capture self-reported global mental distress. The D-Score consists of four items, with each focusing on different aspects of psychological discomfort: depressive symptoms, a sense of meaninglessness, confusion about life, and anxiety about the future. Participants respond to each item using a four-point scale. Higher scores indicated more significant distress. The D-Score has demonstrated robust reliability and validity (McDonald’s Omega = 0.832 in wave 33) [32].

Sleep problems were measured using the validated Jenkins Sleep Scale-4, which was developed to capture common sleep problems over the past month [33]. The JSS-4 comprises four items, each focusing on a different aspect of sleep problems: trouble falling asleep, waking up several times per night, trouble staying asleep, and waking up feeling tired despite a full night’s sleep. Participants indicated how often they experienced each issue using a six-point scale ranging from “never” to “22–31 days”. Higher scores indicated more frequent and severe sleep problems. The Jenkins Sleep Scale-4 has been validated to have good internal consistency (McDonald’s omega = 0.858 in wave 33).

Overall life satisfaction was measured using the Global Satisfaction with Life Scale (G-SLS). Respondents were asked to rate their agreement with the statement, “Taking all together, how do you assess your current life situation? In my life, I am.…” on a 5-point scale ranging from “very satisfied” to “not satisfied at all” [34]. Higher scores indicate lower satisfaction levels. The G-SLS showed good convergent validity and reliability [35].

Self-rated health (SRH) was assessed using a single-item measure that captures participants’ overall perceptions of their health status. SRH was measured using a single-item question: “How would you rate your current health status?” [36,37]. Participants answered on a symmetrical 5-point Likert scale ranging from very good to bad. This measure of SRH is widely used in survey research due to its simplicity and strong predictive validity for various health outcomes, including mortality. SRH has been validated across numerous populations and settings, demonstrating good reliability and robustness as indicators of general health perceptions [38,39].

#### 2.2.2. Covariates—Perceived Threat from Crisis

This study includes four covariates to measure individuals’ perceived threats from various global crises. Each covariate was assessed using a 4-point Likert scale ranging from “weak” to “strong”. The covariates are described as follows.

The “Perceived Threat from Rising Cost of Living” (PT-RCL) covariate evaluated the participants’ perception of the threat posed by the increasing cost of living. The “Perceived Threat from the Consequences of the COVID-19 Pandemic” (PT-COV) measured the level of threat participants experienced due to the ongoing COVID-19 pandemic. The “Perceived Threat from Climate Change” (PT-CLC) covariate assessed participants’ anxiety and concern regarding the impact of climate change. The “Perceived Threat from the Consequences of the Russia-Ukraine War” (PT-RUW) covariate was introduced in wave 33 (2022) to measure the perceived threat from the Russia-Ukraine War. As this covariate is time-specific, PT-RUW is a time-invariant measure, providing insights into the perceived threat at the time of measurement during wave 33 only. In contrast, PT-CLC, PT-COV, and PT-RCL were time-variant covariates assessed in both waves.

### 2.3. Statistical Analysis

Descriptive statistics were used to summarize sample characteristics, outcome variables, and covariates. Dichotomous and categorical variables were described using frequencies (*N*) and percentages (%), whereas continuous variables were expressed as means (*M*) and standard deviations (*SD*).

The primary analysis method applied was a univariable linear mixed-effects model (LMM) using restricted maximum likelihood estimation. This approach allowed for the assessment of the progression of different outcome measures over time and the influence of various covariates. LMMs are particularly suitable for addressing random missing observations and the nonindependence of repeated measurements within individuals. The models were constructed according to Cheng’s guidelines [40]. All statistical analyses were performed in R Studio Version 4.3.2 (R Foundation, Boston, MA, USA) using the following packages: lme4 with restricted maximum likelihood, lmerTest, nlme, MuMIn, r.squaredGLMM, and glmm.hp. The statistical significance level was set at *p* < 0.05.

#### 2.3.1. Unadjusted Analysis

To examine the trends of the five outcome variables and four covariate variables over time, a series of linear mixed-effects models (LMMs) were run for each outcome and covariate. Time was included as a fixed effect and participants were treated as random effects to account for individual variability in repeated measures. The unadjusted analysis offers a broad view of group trends over time, averaging the outcomes across the entire sample. However, this approach can potentially mask the significant effects present at the individual level. This analysis allowed for a comparison of the estimates before and after covariate adjustment.

#### 2.3.2. Adjusted Analysis

Subsequently, a series of adjusted analyses were performed to examine the impact of covariates on the outcome variables over time. For each outcome variable, an LMM was run, including time and four covariates as fixed effects, whereas the intercept remained the only random effect.

These models incorporated three time-varying covariates measured at both time points and one time-invariant covariate, PT-RUW, measured only at wave 33. To enable a meaningful interpretation of the intercept as the expected value of the outcome variable when PT-RUW was at its average value, the time-invariant covariate PT-RUW was grand-mean-centered. All LMMs used a time-coding scheme, where wave 33 was coded as 0 and wave 32 was coded as −1. This decision was based on the temporal nature of the covariates, as it ensured robust data representation and meaningful interpretation of the effects of all included covariates on the outcome variables [41].

#### 2.3.3. Variance Explanation and Hierarchical Partitioning Derived from Adjusted Analysis

The pseudo-R2 method suggested by Nakagawa and Schielzeth [41] was used to estimate the effect size using the variance explained by LMMs. The explained variance was calculated as R^2^ using the r.squaredGLMM function in the MuMIn package [42]. The marginal R^2^ (mR^2^) represents the proportion of the total variance explained by fixed effects only. In contrast, the conditional R^2^ (cR^2^) estimates the variance the entire model explains (both fixed and random effects). Hierarchical partitioning was performed using the glmm.hp package to decompose variance.

The hierarchical partitioning process provides a formal method for estimating the relative contributions or “importance” of various independent predictors in explaining the total outcome variance (R^2^) in the LMM. To further decompose the variance, hierarchical partitioning was performed using the glmm.hp package. As described by Chevan and Sutherland [43], this method provides a systematic approach for estimating the relative contributions or “importance” of various independent predictors to explain the total outcome variance (R^2^) within the LMM framework. The variables were ranked according to their independent explanatory power, allowing for a clearer understanding of the individual impact of each predictor on the overall model. While hierarchical partitioning is becoming more widely used in the ecological literature, e.g., [42,43], its application in health sciences, particularly with LMMs, is relatively novel to the best of our knowledge.

## 3. Results

### 3.1. Descriptive Statistics

Table 2 presents the means, *t*-values, and *p*-values for the outcome variables and covariates for Waves 32 and 33, indicating changes over time.

### 3.2. Unadjusted Analysis: General Trend of Mental Health over Time

Unadjusted LMMs were constructed to investigate general changes in outcome variables and covariates from W32 to W33. These models examine the overall group trend over time without considering individual differences. The results are presented in Table 2. There was a significant increase in mental distress (*p* < 0.001) and sleep problems (*p* < 0.001) during this period. In contrast, changes in physical complaints (*p* = 0.143), life satisfaction, and self-rated health were not statistically significant (*p* > 0.05).

Among the covariates, the perceived threat of rising costs of living increased significantly (*p* < 0.001), while the perceived threat from COVID-19 decreased significantly (*p* < 0.001). No substantial changes were observed in the perceived threat of climate change (*p* > 0.05).

### 3.3. Adjusted Analysis: Individual and General Trends in Mental Health

Adjusted LMMs were constructed to examine the impact of time and perceived threats on the outcome variables. Table 3 presents the results of the adjusted LMMs.

Although no significant temporal effect was observed for physical complaints across the entire sample (*p* > 0.05), individuals reporting higher PT-RCL and PT-COV exhibited significantly increased physical complaints (*p* < 0.05 and *p* < 0.01, respectively), irrespective of the measurement time point. This suggests that although there was no general trend of increasing physical complaints over time, individuals who reported higher levels of PT-RCL and PT-COV at one point also reported more physical complaints at the same time point. Neither PT-CLC nor PT-RUW were significantly associated with physical complaints (both *p* > 0.05).

The analysis revealed a significant temporal effect of mental distress across the entire sample (*p* < 0.001). Additionally, at the individual level, the PT-RCL and PT-COV were significantly associated with increased mental distress (*p* = 0.005 and *p* < 0.001, respectively). This suggests a dual trend, characterized by a general increase in mental distress over time across the sample. At the individual level, those who reported higher levels of PT-RCL and PT-COV also reported higher levels of mental distress at the same time point.

The analysis revealed a significant temporal effect on sleep problems (*p* < 0.001). In addition, individual-level factors were significantly associated with sleep problems. Specifically, a higher PT-RCL (*p* < 0.05), PT-COV (*p* < 0.001), and PT-CLC (*p* < 0.01) were significantly associated with increased sleep problems. The findings elucidate a bidirectional pattern characterized by a general increase in sleep problems over the study period across the sample; at the individual level, those who reported higher levels of perceived threats consistently exhibited elevated sleep disturbances at both time points, in addition to the general trend. Notably, PT-RUW did not have a significant effect on sleep problems (*p* > 0.05).

The analysis revealed no significant temporal effect on life satisfaction across the entire sample (*p* > 0.05). However, at the individual level, higher PT-RCL and PT-COV scores were significantly associated with decreased life satisfaction (*p* < 0.001 for both). This suggests that while there was no general trend of changing life satisfaction over time, individuals who reported higher levels of PT-RCL and PT-COV also reported lower life satisfaction at that same time point. Notably, PT-CLC and PT-RUW did not demonstrate statistically significant effects on life satisfaction (both *p* > 0.05).

Similarly, no significant temporal effect was observed for self-rated health across the entire sample (*p* > 0.05). At the individual level, only PT-COV was significantly associated with poorer self-rated health (*p* < 0.05). This suggests that while there was no general trend of changing self-rated health over time, individuals who reported higher levels of PT-COV also reported poorer self-rated health at that same time point. The effects of PT-RCL, PT-CLC, and PT-RUW on self-rated health were not significant (all *p* > 0.05).

### 3.4. Total Variance Explained and Decomposition of Explained Variance with Hierarchical Partitioning

Table 3 also reports the marginal R^2^ (mR^2^) and conditional R^2^ (cR^2^) values for each model. The mR^2^, which represents the variance explained solely by fixed effects, ranged from 0.026 in the self-rated health model to 0.145 in the life satisfaction model, indicating a modest contribution of fixed effects to the explained variance. Conversely, the cR^2^ values, including fixed and random effects, ranged from 0.522 in the Life Satisfaction model to 0.703 in the Mental Distress model, suggesting that random effects accounted for a substantial portion of the explained variance.

**Figure 3:**
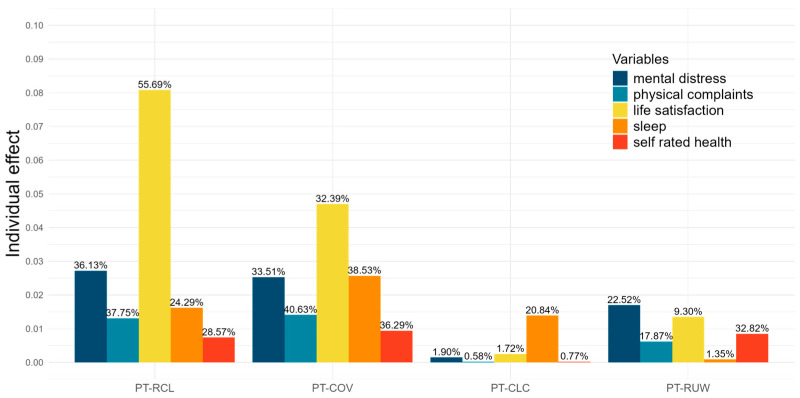
The relative importance of covariates on outcome variables based on hierarchical partitioning analysis.

Furthermore, hierarchical variance partitioning analysis was conducted to determine the relative importance of the predictor variables for the outcome variables. The results are shown in Figure 3. In the life satisfaction model, the fixed effects (predictor variables) accounted for 14.5% of the total variance (mR^2^ = 0.145). Among these predictors, PT-RCL and PT-COV were the strongest, explaining 8% and 4% of the total variance in life satisfaction, respectively. The other predictors in the life satisfaction model and all predictors in the other outcome models explained less than 4% of the total variance, suggesting that they played a relatively minor role in influencing mental health and well-being outcomes.

When considering the distribution of the explained variance among the predictors within each outcome model, PT-RCL and PT-COV emerged as the most influential factors. According to the physical complaints model, the PT-RCL and PT-COV explained 38% of the variance. Furthermore, hierarchical variance partitioning analysis was conducted to determine the relative importance of the predictor variables for the outcome variables. The results are summarized in Figure 3.

According to the life satisfaction model, PT-RCL and PT-COV were the strongest predictors, explaining 56% and 32% of the marginal variance, respectively. The other predictors in the life satisfaction model explained less than 9% of the marginal variance. According to the physical complaints model, PT-RCL and PT-COV were the most influential predictors, explaining 38% and 41% of the marginal variance, respectively. Similarly, in the mental distress model, PT-RCL and PT-COV remained the strongest predictors, contributing 36% and 34% of the explained marginal variance, respectively. For Sleep Problems, PT-COV was the most prominent predictor, explaining 38.53% of the variance, followed by PT-RCL (24%) and PT-CLC (20%). According to the life satisfaction model, PT-RCL and PT-COV were again identified as the most influential predictors, with 56% and 32% contributions, respectively. Finally, in the self-rated health model, PT-COV exhibited a modest contribution of 36%, with the contributions of the other predictors being notably smaller.

## 4. Discussion

To our knowledge, this study is the first to examine the compounded impacts of multiple global crises, including climate change, the COVID-19 pandemic, the Russia-Ukraine War, and rising living costs, on various aspects of mental health among a representative sample of East German citizens from 2021 to 2022. Our findings provide a nuanced understanding of how these concurrent crises affect mental health outcomes at both group and individual levels. These findings provide several vital insights. The data showed significant increases in mental distress and sleep difficulties from 2021 to 2022. Our longitudinal mixed-model analyses, which included both unadjusted and adjusted models, allowed us to distinguish between overall trends and individual-level effects.

Unadjusted analyses examining overall group trends showed significant increases in mental distress and sleep difficulties from 2021 to 2022. However, they reported no significant changes in physical complaints, life satisfaction, or self-rated health at the group level over time. In contrast, the adjusted analyses, which accounted for individual-level perceptions of threat, revealed more complex patterns.

Specifically, the adjusted analyses indicated that while there was no significant temporal effect on physical complaints, life satisfaction, or self-rated health across the entire sample, individual-level factors such as PT-RCL and PT-COV were significantly associated with these outcomes. Interestingly, only PT-COV was significantly associated with self-rated health. This suggests that although the group as a whole did not show significant changes over time, individuals who perceived higher levels of these threats consistently reported worse outcomes in these areas. For mental distress and sleep problems, adjusted analyses revealed significant temporal effects, indicating a general increase in these issues over time across the sample. Additionally, higher PT-RCL and PT-COV levels were significantly associated with increased mental distress and sleep problems at the individual level. This dual trend suggests that while the entire sample experienced worsening mental distress and sleep problems over time, individuals with higher perceived threats reported even greater levels of these issues, adding to the general temporal effect. Changes in PT-CLCs only contributed significantly to worsened sleep problems, although their impact on other health outcomes was not significant. Notably, PT-RUW did not significantly affect any of the mental health outcomes measured, suggesting that immediate economic and health-related stressors have a more pronounced impact on psychological well-being than more distant and abstract threats such as climate change or geopolitical conflicts. Our findings suggest a potential contrast: while PT-RCL appeared to significantly impact various mental health indicators, PT-RUW showed no significant effects in our sample. This discrepancy might be noteworthy when considered alongside media coverage in Germany, where the Russia–Ukraine War often dominates public discourse [44]. Our results could indicate that despite media focusing on the war, immediate economic pressure may have a greater impact on individuals’ mental well-being.

Moreover, the lack of significant changes in physical complaints, life satisfaction, and self-rated health over time suggests that these aspects of well-being may be more resilient or slower in manifesting changes in response to global crises. The stable measures of life satisfaction and self-rated health observed in this study suggest that while individuals may experience heightened mental distress and sleep problems, their broader perception of life quality and health status remains relatively intact over a shorter period [45]. However, the adjusted analyses revealed that these outcomes are affected by individual perceptions of threat, particularly economic instability and the pandemic.

Furthermore, the observed changes in the perceived threat of all four global crises align with the findings from the Security Report in 2021 and 2023 [8,9], indicating a substantial increase in concerns about inflation and rising living costs from 2021 to 2023, mirrored by the significant increase in PT-RCL observed in this study. Similarly, the decrease in PT-COV aligns with the sharp decline in apprehensions regarding COVID-19 reported in the Security Report from 2021 to 2023.

The findings of this study are consistent with those of previous studies, highlighting the adverse effects of global crises on mental health. The significant associations between PT-RCL and PT-COV and increased physical complaints, mental distress, and sleep problems, as well as decreased life satisfaction, corroborate previous findings on the negative impact of financial insecurity and pandemic-related stress on mental health [15,46]. Similarly, the link between PT-CLC and worsened sleep problems is consistent with research demonstrating a relationship between climate change-related negative emotions and insomnia symptoms [14].

However, the non-significant effect of PT-RUW on mental health outcomes contrasts with earlier studies, such as Mărcău et al. [47], who found a robust negative correlation between worries about the ongoing war and the overall quality of life in the general Romanian population. Conversely, our findings align more closely with those of Scharbert et al. [23], who observed an acute decline in well-being in European countries on the day of the Russian invasion, followed by recovery to previous levels over the following weeks. This discrepancy may be attributed to differences in study design, sample characteristics, and timing of assessments relative to the onset of the war.

The varying results regarding the impact of these crises, particularly PT-RUW, on mental health outcomes, may be related to the study’s investigation of multiple simultaneous crises. Previous studies have examined the impact of a single crisis on mental health, which may have yielded misleading results. The influence of a third variable, rather than a direct causal relationship, could make the results more significant. However, this hypothesis requires further investigation.

### Limitations

Although our study provides valuable insights into the effects of multiple global crises on mental health, several limitations should be acknowledged. First, our approach to examining compounded effects primarily focused on the sum of the main effects of covariates, which may oversimplify the true complexity of how multiple crises interact and impact mental health. The potential synergistic effects and interactions between different perceived threats were not fully explored in our model. Moreover, the true compounded effect could extend beyond the sum of individual effects, as the whole may be greater than the sum of its parts in the context of mental health impact. Although including two-way interactions would have allowed for a more nuanced understanding, it would have significantly increased model complexity, potentially compromising model stability and increasing the risk of overfitting, given our sample size [48]. These limitations highlight the challenges of fully capturing the intricate dynamics of how multiple global crises collectively impact mental health.

A notable observation of this study is the substantial discrepancy between the marginal and conditional R^2^ values across models. mR^2^, representing the variance explained solely by fixed effects, was relatively modest, ranging from 0.026 to 0.145 between the models. By contrast, the cR^2^ values, which account for fixed and random effects, were much more remarkable, ranging from 0.522 to 0.703. This gap indicates that random effects, which capture individual and unmeasured factors, play a substantial role in explaining the variance in the mental health outcomes in this study. More comprehensive models including a broader range of variables are required to fully capture these nuances.

Another potential factor contributing to the large discrepancy between the mR^2^ and cR^2^ values may be the use of short scales with few items to measure the complex mental health constructs in our study.

Short scales may exhibit reduced variance, lower reliability, and potential validity issues because they do not adequately capture all facets of a construct [49]. This reduced variance and reliability can limit the capacity of the study to detect nuanced changes and potentially lead to an underestimation of the true impact on mental health outcomes. However, the brevity of these scales also has advantages, such as making data collection more economical and likely reducing participant dropout rates [50].

Finally, reliance on self-reported measures may be subject to response bias and social desirability bias. Furthermore, the study sample was limited to a representative age cohort born in 1973 in the former German Democratic Republic, which limits the generalizability of the findings to other age groups and populations. Moreover, the relatively short timeframe between the two waves of data collection (2021–2022) may not capture the long-term effects of global crises. Additionally, the study did not account for potential confounding factors such as pre-existing mental health conditions, socioeconomic status, or individual coping mechanisms. Future research should address these limitations and employ more comprehensive and detailed measures to capture the complexity of these constructs more accurately. This approach would enhance the reliability of the data and improve the capacity of the study to detect nuanced changes and associations with global crises, thus providing a more thorough understanding of the impacts of mental health. Moreover, the current findings can be extended by examining the long-term consequences of these global stressors and identifying the protective factors that promote resilience in the face of adversity. It is vital to emphasize that our statistical analyses focused primarily on the direct consequences of perceived threats on mental health outcomes. Although this method offers valuable information regarding the overall impact of these threats, it does not enable us to examine how these effects may change over time. To explore the potential temporal dynamics of these relationships, future research should incorporate the interaction effects between time and perceived threats into the models. This would allow us to determine whether the influence of perceived threats on mental health outcomes intensifies, diminishes, or remains stable over time. By incorporating interaction effects between time and perceived threats into future research, we can gain a more comprehensive understanding of the complex interplay between global crises and mental health over time. Insight into these temporal dynamics is crucial for developing more effective and targeted interventions, focusing on either immediate support or long-term coping strategies. If economic instability and health-related fear are critical drivers of psychological distress, as suggested in the present study, effective interventions targeting these stressors should be explored. Mental health professionals and policymakers should prioritize efforts to mitigate the adverse effects of economic instability and crises on mental health.

## 5. Conclusions

This study provides valuable insights into the compounded effects of multiple global crises on the mental health of East German adults. Our findings highlight that economic instability and pandemic-related concerns are key drivers of psychological distress, while more distant threats, such as climate change and geopolitical conflicts, may have a less immediate impact. The discrepancies between unadjusted and adjusted analyses emphasize the complex interplay between group-level trends and individual-level perceptions in shaping mental health outcomes during intersecting global crises, which has important implications for mental health professionals and policymakers. Efforts to mitigate the adverse effects of economic instability and health-related crises on mental health should be prioritized. Future research should address these limitations by incorporating more comprehensive measures, exploring the interaction effects between perceived threats and time, and examining the long-term consequences of global stressors. While our approach of examining multiple crises simultaneously may not capture all the complexities of their interactions, it represents a significant step toward understanding their compounded effects on mental health. This study may lay the foundation for future investigations into the complex landscape of global challenges and their impact on psychological well-being, ultimately contributing to the development of more effective and targeted interventions for supporting mental health during periods of multiple overlapping global crises.

## Figures and Tables

**Table 1:** Sociodemographic characteristics of the sample.

Characteristic	Value
Gender (in %)	
Male	45.5
Female	54.5
Age (mean)	49.9
Children (in %)	80.4
Number of children (mean)	1.8
Family status (in %)	
Married, living together	57.0
Married, living separately	3.5
Single	27.2
Divorced	10.1
Widowed	2.2
Steady Partner	81.8
Occupational Status (in %)	
Worker	18.8
Employee	62.1
Self-employed	9.4
Housewife/Househusband	0.6
Civil servant	5.0
Unemployed	1.9
Other	2.2
Net household Income in € (median)	3500 to 3999
Current Residence (in %)	
Lives in West Germany	21.3
Lives abroad	2.8
Lives in East Germany	75.8

Note. *N* = 319 measured in 2022 (W33). The median value is presented as a range because it is derived from a categorical variable.

**Table 2:** Descriptive statistics and results of unadjusted LMMs.

Variable	W32 (2021)	W33 (2022)	*t*	*p*
physical complaints, mean, (0–12)	3.56	3.76	1.47	0.143
mental distress, mean, (0–8)	1.07	1.29	3.22	0.001 **
sleep problems, mean, (0–20)	4.87	6.27	3.91	<0.001 ***
Life Satisfaction, mean, (0–4)	1.28	1.34	1.16	0.249
Self-rated health, mean, (0–4)	1.42	1.47	0.78	0.436
PT-RCL, mean, (0–3)	1.95	2.44	12.49	<0.001 ***
PT-COV, mean, (0–3)	1.86	1.46	−6.44	<0.001 ***
PT-CLC, mean, (0–3)	1.77	1.71	−1.55	0.123
PT-RUW, mean, (0–3)		2.09		

Note. ** *p* < 0.01. *** *p* < 0.001.

**Table 3:** Fixed effects, mR^2^, and cR^2^ of the adjusted linear mixed covariate models (*N* = 319).

		Estimates	*SE*	*t*	*p*
Physical complaints	time	0.212	0.135	1.571	0.117
	covariate PT-RCL	0.308	0.155	1.983	0.048 *
	covariate PT-COV	0.308	0.119	2.589	0.010 **
	covariate PT-CLC	0.038	0.133	0.289	0.773
	covariate PT-RUW	0.137	0.201	0.684	0.495
	marginal R^2^	0.035			
	conditional R^2^	0.681			
Mental distress	time	0.294	0.086	3.410	<0.001 ***
	covariate PT-RCL	0.284	0.010	2.844	0.005 **
	covariate PT-COV	0.269	0.076	3.530	<0.001 ***
	covariate PT-CLC	0.030	0.086	0.352	0.725
	covariate PT-RUW	0.180	0.130	1.389	0.165
	marginal R^2^	0.075			
	conditional R^2^	0.703			
Sleep problems	time	1.172	0.288	4.074	<0.001 ***
	covariate PT-RCL	0.631	0.319	1.976	0.049 *
	covariate PT-COV	0.818	0.243	3.361	<0.001 ***
	covariate PT-CLC	0.651	0.270	2.412	0.016 *
	covariate PT-RUW	−0.132	0.387	−0.341	0.733
	marginal R^2^	0.067			
	conditional R^2^	0.691			
Life satisfaction	time	−0.079	0.005	−1.551	0.122
	covariate PT-RCL	−0.298	0.051	−5.882	<0.001 ***
	covariate PT-COV	−0.170	0.041	−4.182	<0.001 ***
	covariate PT-CLC	0.041	0.041	0.993	0.321
	covariate PT-RUW	0.001	0.056	0.011	0.991
	marginal R^2^	0.145			
	conditional R^2^	0.522			
Self-rated health	time	−0.038	0.046	−0.820	0.413
	covariate PT-RCL	−0.059	0.057	−1.163	0.245
	covariate PT-COV	−0.082	0.039	−2.072	0.039 *
	covariate PT-CLC	0.012	0.043	0.244	0.807
	covariate PT-RUW	−0.074	0.063	−1.187	0.236
	marginal R^2^	0.026			
	conditional R^2^	0.622			

Note. PT-RCL = perceived threat from rising living costs; PT-COV = perceived threat from the COVID-19 pandemic; PT-CLC = perceived threat from climate change; PT-RUW = perceived threat from the Russia-Ukraine War. * *p* < 0.05. ** *p* < 0.01. *** *p* < 0.001.

## Data Availability

The analysis was based on the Saxon Longitudinal Study. The data, as well as all questionnaires with their individual items of the Saxon Longitudinal Study, are archived at the Leibniz Institute for the Social Sciences (gesis) and can be obtained for research purposes at https://doi.org/10.4232/1.13875, https://doi.org/10.4232/1.14147.
